# Novel Ex Vivo Human Osteochondral Explant Model of Knee and Spine Osteoarthritis Enables Assessment of Inflammatory and Drug Treatment Responses

**DOI:** 10.3390/ijms19051314

**Published:** 2018-04-28

**Authors:** Jeroen Geurts, Doria Jurić, Miriam Müller, Stefan Schären, Cordula Netzer

**Affiliations:** 1 Department of Spine Surgery, University Hospital of Basel, 4031 Basel, Switzerland; Stefan.Schaeren@usb.ch (S.S.); Cordula.Netzer@usb.ch (C.N.); 2 Department of Biomedical Engineering, University Hospital of Basel, 4123 Allschwil, Switzerland; doria.j@hotmail.com (D.J.); MiriamMWeil@web.de (M.M.); 3 Institute for Chemistry and Bioanalytics, University of Applied Sciences and Art Northwestern Switzerland, 4132 Muttenz, Switzerland

**Keywords:** osteoarthritis, osteochondral, experimental model, inflammation, bone metabolism, knee, spine

## Abstract

Osteoarthritis of the knee and spine is highly prevalent in modern society, yet a disease-modifying pharmacological treatment remains an unmet clinical need. A major challenge for drug development includes selection of appropriate preclinical models that accurately reflect clinical phenotypes of human disease. The aim of this study was to establish an ex vivo explant model of human knee and spine osteoarthritis that enables assessment of osteochondral tissue responses to inflammation and drug treatment. Equal-sized osteochondral fragments from knee and facet joints (both *n* = 6) were subjected to explant culture for 7 days in the presence of a toll-like receptor 4 (TLR4) agonist and an inhibitor of transforming growth factor-beta (TGF-β) receptor type I signaling. Markers of inflammation, interleukin-6 (IL-6) and monocyte chemoattractant protein-1 (MCP-1), but not bone metabolism (pro-collagen-I) were significantly increased by treatment with TLR4 agonist. Targeting of TGF-β signaling resulted in a strong reduction of pro-collagen-I and significantly decreased IL-6 levels. MCP-1 secretion was increased, revealing a regulatory feedback mechanism between TGF-β and MCP-1 in joint tissues. These findings demonstrate proof-of-concept and feasibility of explant culture of human osteochondral specimens as a preclinical disease model, which might aid in definition and validation of disease-modifying drug targets.

## 1. Introduction

Osteoarthritis (OA) is a chronic disorder involving movable joints that is characterized by cell stress and extracellular matrix degradation initiated by micro- and macro-injury that activates maladaptive repair responses including pro-inflammatory pathways of innate immunity [1]. The prevalence of radiographic OA is high in facet joints of the lumbar spine and the knee joints of elderly individuals, and is associated with age and obesity [2,3,4]. While the etiopathogenesis of OA still remains unknown, it has been established that pathological changes to several tissues including articular cartilage, synovium and subchondral bone and marrow are involved in joint degeneration [5,6,7]. The presence of synovial inflammation and bone marrow lesions is strongly associated with the progression of knee and facet joint osteoarthritis in humans [8,9,10]. OA severity is correlated with increased expression of a number of pro-inflammatory mediators, including interleukin-6 (IL-6) and monocyte chemoattractant protein-1 (MCP-1/CCL2) [11,12]. It has been shown in experimental and human OA that agonists of Toll-like receptor 4 (TLR4), such as lipopolysaccharide (LPS) and damage-associated molecular patterns (DAMPs) produced in the degenerated joint, play a central role in the inflammatory response in diseases joints [13,14,15,16]. Nevertheless, a disease-modifying OA drug (DMOAD) is still lacking, and total joint replacement remains the standard symptomatic treatment for end-stage disease. 

Definition of novel drug targets and preclinical evaluation of DMOADs predominantly relies on the use of in vitro models, including isolated chondrocytes and osteoblasts from human OA tissues, or experimental murine models [17,18]. Animal models have demonstrated promising results for therapeutic treatment of knee OA based on inhibition of transforming growth factor-β1 (TGF-β1) in subchondral bone, and Adamts5 or WNT/β-catenin signaling in articular cartilage [19,20,21]. However, these findings have not yet resulted in a DMOAD therapy for OA in humans. Several limitations of in vitro and experimental models pose serious challenges to the translation of preclinical findings into clinical practice. While different clinical phenotypes are known in humans, including inflammatory, metabolic and biomechanical OA [22], experimental models are predominantly surgically induced post-traumatic OA [23]. The vast majority of in vivo models are focused on knee OA, and validated models for spine, hand and hip OA are lacking. In addition, the relatively small size of murine joints complicates the assessment of bone marrow lesions and synovitis by magnetic resonance imaging, which are important diagnostic and prognostic imaging biomarkers in human OA [6]. Experimental in vitro studies with isolated chondrocytes and osteoblast provide valuable insight into cellular responses, but do not take the crosstalk between different joint tissues into account. Tissue culture models of isolated articular cartilage from OA specimens have proven valuable in functional studies and detection of pathological hallmarks [17,18]. There is a paucity of models that focus on additional joint compartments, including subchondral bone and marrow tissue. The challenges for future DMOAD development include recognition of OA as a complex disease with multiple phenotypes and potential joint-specific pathomechanisms [24].

The aim of our study was to establish an ex vivo osteochondral tissue culture model of human knee and facet joint OA that is responsive to an inflammatory challenge and enables the assessment tissue responses to drug treatment effects. Treatment with a TLR4 agonist led to upregulated secretion of IL-6 and MCP-1 proteins, while leaving bone metabolism, assessed by pro-collagen-I (pro-Col-I), unaffected. Inhibition of TGF-β receptor type I signaling significantly reduced pro-Col-I and IL-6 secretion in knee and facet joint specimens, but led to increased MCP-1 levels. These findings provide proof-of-concept and feasibility of explanted osteochondral clinical specimens as preclinical human and joint-specific OA model. 

## 2. Results

### 2.1. Tissue Viability after Explant Culture

**Figure 1:**
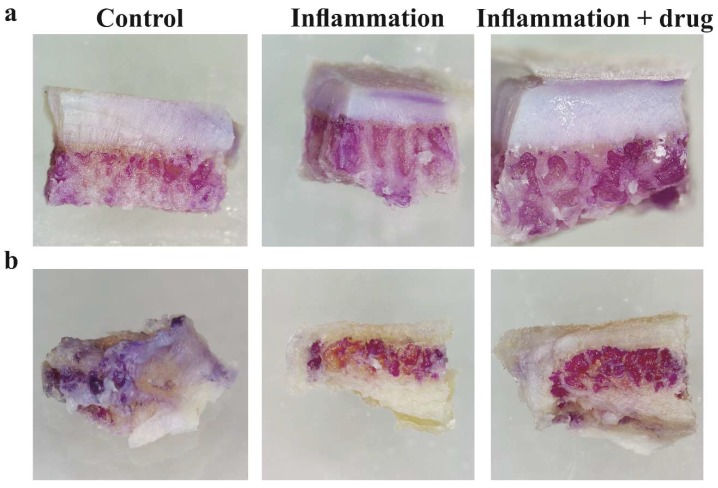
Tissue viability after explant culture of osteochondral specimens from (**a**) knee and (**b**) facet joint osteoarthritis. Fresh clinical specimens were cut in equal-sized fragments and cultured in osteogenic culture medium for one week. Samples were either left untreated (control) or challenged with 1 μg/mL lipopolysaccharide (inflammation) in the absence and presence of a drug treatment (10 μM TGF-β receptor type I inhibitor).

Osteochondral specimens were prepared from osteoarthritic knee tibial plateaus or facet joints. Cancellous bone from iliac crest and distal lateral tibial plateau served as osteal tissue controls. Samples were cultured in osteogenic culture medium with and without an inflammatory stimulus and drug treatment, and tissue viability was evaluated by assessment of cell metabolic activity using MTT staining (Figure 1). Viable cells were readily detected in subchondral bone marrow and cartilage tissues. Gross evaluation of staining patterns and intensity revealed no reduced tissue viability under inflammatory or drug treatment conditions.

### 2.2. Secretion of Pro-Collagen-I and Inflammatory Mediators under Basal and Inflamed Conditions

**Figure 2:**
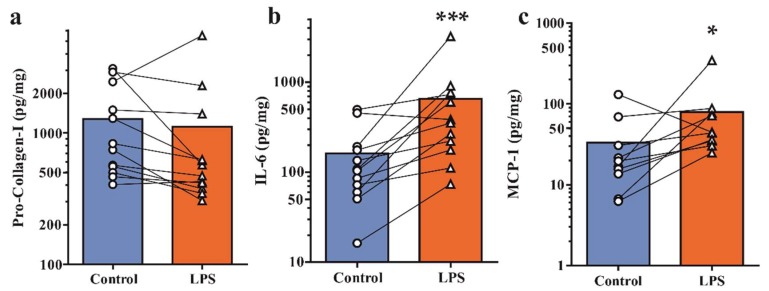
Assessment of secreted markers of bone metabolism and inflammation under basal and inflammatory conditions. Osteoarthritic specimens were left untreated in osteogenic culture medium (control) or challenged with LPS. Secreted protein levels of (**a**) pro-Col-I, (**b**) IL-6 and (**c**) MCP-1 were determined by ELISA. * *p* < 0.05, *** *p* < 0.001 by ratio paired *t*-test.

Protein levels of pro-collagen-I (pro-Col-I), as marker of bone metabolism, and inflammatory cytokine (IL-6) and chemokine (MCP-1) were determined in tissue-conditioned medium by ELISA. All specimens secreted pro-Col-I, IL-6 and MCP-1 under basal conditions (Table 1). Weight-normalized expression levels of osteal tissues only were approximately tenfold higher than osteoarthritic osteochondral specimens. Next, we assessed whether explanted tissues specimens were responsive to an inflammatory insult. Samples were challenged with Toll-like receptor 4 agonist lipopolysaccharide (LPS, 1 μg/mL), which has been found in synovial fluid of inflamed OA knee joints and mimics signaling induced by damage-associated molecular patterns that are present in degenerative joints [13,14,15,16]. Pro-Col-I expression was unaffected by LPS challenge in osteoarthritic specimens (Figure 2a). Protein levels of the inflammatory mediators IL-6 and MCP-1 were respectively 4-fold and 2.4-fold upregulated in knee and facet joints (Figure 2b,c). A similar response was observed in osteal tissue controls, which showed unaffected pro-Col-I levels and 1.5- and 2.4-fold upregulation of IL-6 and MCP-1, respectively. These findings demonstrate that osteochondral tissue specimens are capable of an inflammatory cytokine response under ex vivo culture conditions.

### 2.3. Inhibition of TGF-β Receptor Type I Signaling Modulates Bone Metabolism and Inflammatory Mediators

**Figure 3:**
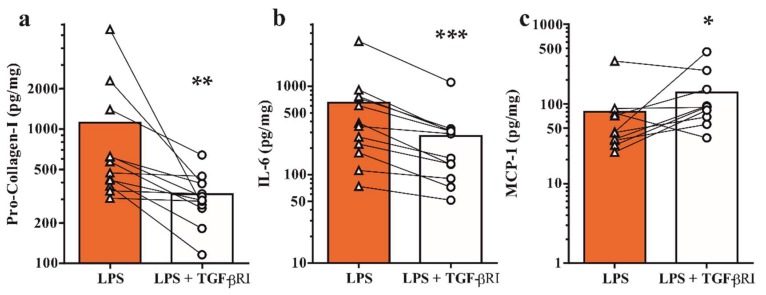
Assessment of secreted markers of bone metabolism and inflammation under inflammatory conditions in the presence and absence of TGF-β receptor type I inhibition. Osteoarthritic specimens were challenged with LPS (1 μg/mL) and treated with 10 μM SB-505124. Secreted protein levels of (**a**) pro-Col-I, (**b**) IL-6 and (**c**) MCP-1 were determined by ELISA. * *p <* 0.05, ** *p <* 0.05, *** *p <* 0.001 by ratio paired *t*-test.

Next, we sought to evaluate whether drug treatment of explanted tissues would lead to a measurable effect. As proof of concept we investigated the effects of pharmacological inhibition of TGF-β receptor type I signaling, which has been described as a pivotal signaling pathway in joint homeostasis and osteoarthritis [20,25,26]. Osteochondral specimens were challenged with LPS in the presence of 10 μM SB-505124 and pro-Col-I, IL-6 and MCP-1 levels were determined by ELISA. Targeting of TGF-β signaling led to a significant reduction in bone metabolism, as demonstrated by a 3.4-fold reduction of pro-Col-I secretion (Figure 3a). Similarly, IL-6 levels were found to be 2.4-fold reduced (Figure 3b). In contrast, MCP-1 expression was 1.7-fold upregulated in the presence of a TGF-β receptor type I inhibitor (Figure 3c). Subgroup analysis of osteoarthritic knee and facet joints and osteal tissues revealed differences in treatment effects (Table 2). Notably, levels of bone metabolism and inflammatory markers were significantly altered in osteoarthritic specimens, but not osteal tissues only. In addition, upregulation of MCP-1 protein expression was significant in knee, but not facet joint osteoarthritis. Together, these findings demonstrate that tissue responses of osteochondral specimens from osteoarthritic joints to inflammation and drug treatment can be monitored in an ex vivo explant model.

## 3. Discussion

The development of a DMOAD remains an unmet clinical need in the treatment of OA in humans. Despite encouraging results from experimental OA models [19,20,21], the translation of preclinical studies to clinical practice has proved to be challenging. Selection of an appropriate model that accurately reflects the joint-specific pathomechanisms and clinical phenotypes observed in human disease is crucial for successful DMOAD development. Given that experimental murine models are predominantly focused on post-traumatic knee joint OA, we sought to establish a novel human ex vivo OA model based on explant culture of clinical specimens from knee and spine. Our findings showed that human osteochondral tissues could readily be cultured without appreciable loss of viability. Explanted specimens were capable of mounting an inflammatory response to a TLR4 agonist, which mimics signaling induced by endogenous ligands produced in the degenerative joint. Inhibition of TGF-β signaling, which is pivotal in OA subchondral bone remodeling [20], reduced bone metabolism and cytokine expression. Upregulation of MCP-1 secretion was uncovered as a potential undesirable side effect of TGF-β signaling inhibition.

To the best of our knowledge, this is the first study using intact osteochondral tissue from human OA specimens as a model for evaluating drug treatment responses. Human and mouse bone and cartilage explants have been used to investigate catabolic responses to stimulation with pro-inflammatory mediators [27,28]. Osteochondral explants from OA patients stimulated for 7 days with IL-17A and TNF-α showed a two- to five-fold increase of cytokine (IL-6) and chemokine (IL-8) secretion and loss of bone volume [27]. Treatment of explanted whole femoral heads with TNF-α and oncostatin-M for 10 days led to increased levels of Col-I resorption marker in conditioned medium [28]. Markers of bone formation, such as alkaline phosphatase, osteocalcin or pro-Col-I, were not assessed in these models. While we used a different stimulus in this study, our results confirm that an inflammatory challenge elicits an innate immune response in osteochondral tissues. Notably, pro-Col-I as a marker of bone formation and metabolism seemed unaffected under inflammatory conditions. Assessment of secreted markers of bone resorption (CTX-I or tartrate-resistant acid phosphatase) or micro-computed tomography of cancellous bone volume could provide insight whether triggering of TLR4 signaling leads to elevated bone resorption in human knee and spine OA. Interestingly, previous histological studies demonstrated high osteoclast activity in subchondral marrow tissues of knee, but not facet joint OA [29,30]. 

Pleiotrophic effects of growth factor and cytokine signaling in different joint tissues are important to consider in DMOAD development. TGF-β receptor type I signaling orchestrates pathological bone formation in experimental OA [20], yet promotes chondrocyte anabolism in human and murine osteoarthritic cartilage tissues [26]. TGF-β1 stimulates the expression and secretion or pro-Col-I in primary human osteoarthritic osteoblasts [31]. While the effects of SB-505124 treatment on cartilage metabolism have not been investigated in this study, we uncovered upregulated MCP-1 secretion under inflammatory conditions as potential undesirable side effect of TGF-β signal pathway inhibition. A regulatory negative feedback loop between MCP-1 and TGF-β1 has previously been demonstrated in kidney tissue [32]. Conversely, a positive regulatory mechanism has been described in blood vessels [33]. Our results suggest that TGF-β signaling in osteochondral tissues reduces MCP-1 protein levels. An important role for MCP-1 signaling in mediating monocyte recruitment, inflammation and cartilage destruction in experimental OA [12] suggest that treatment strategies based on targeting of TGF-β signaling should be carefully evaluated. It should be noted that beneficial treatment effects were obtained by local delivery of neutralizing TGF-β antibody into subchondral bone of a rat OA model [20].

Given the ample evidence for the involvement of crosstalk between cartilage and subchondral bone and marrow tissues in OA joints [34], it is straightforward that systemic treatment strategies targeting a specific tissue compartment need to be screened for side effects. We found increasing normalized secretion levels of pro-Col-I, IL-6 and MCP-1 in osteal tissue specimens, which contain a relatively high marrow to bone tissue fraction. It is therefore likely that expression of the aforementioned markers stems primarily from resident cells of bone (osteoblasts) and marrow (macrophages, stromal cells). Importantly, striking histological differences between clinical knee OA phenotypes and knee, spine and ankle joint OA have been described for subchondral bone marrow tissues [29,30,35,36]. The established explant model might greatly aid in evaluating whether differential inflammatory and treatment responses occur in different joints or clinical phenotypes.

Future research efforts could include the analysis of cartilage catabolic and anabolic markers such as cartilage oligomeric matrix protein, aggrecan or collagen type II fragments. In addition, it would be interesting to determine expression patterns of secreted proteins that have been described to be differentially regulated in OA tissues, such as DKK-1 and sclerostin [37,38]. Stratification of clinical specimens prior to explant culture using MRI-based assessment of joint inflammation and bone marrow lesions might aid in selectively studying clinical phenotypes [8,39].

We acknowledge some limitations of the present study. Specimens were cultured in osteogenic medium containing dexamethasone to sustain activity of bone tissues. Dexamethasone is, however, a corticosteroid with broad anti-inflammatory effects, and inflammatory tissue responses to LPS might therefore have been partially dampened. Nevertheless, we observed a clear inflammatory response, and expression of inflammatory mediators might only be increased when omitting dexamethasone from the culture medium. Furthermore, the explant model focuses on osteochondral tissue responses in an artificial setting. The role of mechanical loading, angiogenesis or crosstalk with synovial tissue and fluid is cannot be considered under the described culture conditions. The influence of synovial inflammation on osteochondral tissues could, however, be investigated by co-culture experiments or stimulation with conditioned medium. Adaptation of the explant model to a mechanical loading bioreactor commonly used for 3D-tissue engineering constructs might enable the evaluation of tissue responses under physiological and pathological joint loading conditions. 

In conclusion, we have provided proof-of-concept and feasibility of an explant culture of human osteochondral clinical specimens from knee and facet joint OA for the evaluation of tissue responses to inflammation and drug treatment. Activation of LPS signaling, mimicking TLR4-induced inflammation mediated by DAMPs, resulted in an inflammatory cytokine response in osteochondral tissues. Inhibition of TGF-β signaling, a key pathway in bone metabolism, modulated pro-Col-I secretion and differentially regulated inflammatory mediators IL-6 and MCP-1. This preclinical disease model may be valuable in defining and validating DMOAD targets in specific joints and clinical phenotypes.

## 4. Materials and Methods

### 4.1. Collection of Clinical Specimens

Five knee tibial plateaus were obtained from patients undergoing total joint arthroplasty (average age 72 ± 5.7 years). Six facet joint specimens were harvested by facetectomy from patients undergoing spine fusion surgery due to lumbar spinal stenosis (average age 74 ± 5.9 years). Iliac crest cancellous bone was obtained as leftover autologous bone graft material from three patients undergoing spine fusion surgery (average age 65 ± 8.4 years). Cancellous bone from the distal portion of non-lesional lateral tibial plateau from two patients (age: 57 and 75 years). Written informed consent was obtained from all patients, and the study protocol was reviewed and approved by the Ethics Committee Northwest and Central Switzerland (No. 147/12, approved 31 August 2012).

### 4.2. Explant Culture of Osteochondral and Osteal Tissue Specimens

Specimens were processed immediately after surgical resection and gently rinsed in sterile phosphate-buffered saline (PBS) to remove blood. Degenerative facet joints, the central portion of the cartilage lesion on tibial plateaus (5 medial, one lateral), osteal tissues were cut into equal-sized samples (50–500 mg wet weight) with a scalpel. Fragments were placed in 8 mL osteogenic culture medium in 6-well plates (αMEM supplemented with antibiotics, 10% fetal bovine serum, 10 mM HEPES, 4 mM l-glutamine, 10^−7^ M dexamethasone, 50 μM l-ascorbic acid-2-phosphate and 10 mM sodium β-glycerophosphate pentahydrate (Sigma-Aldrich, Buochs, Switzerland). Specimens were cultured for one week at 37 °C in a humidified atmosphere containing 5% CO_2_.

### 4.3. Inflammatory Challenge and TGF-β Receptor Type I Inhibitor Treatment

Controls were treated with vehicle (6 μL DMSO) and 16 μL PBS at days 0 and 3. To elicit an inflammatory response, specimens were treated with vehicle and 16 μL of a 500× stock solution of lipopolysaccharides (LPS) from *Escherichia coli* O111:B4 (L2630 Sigma-Aldrich, final concentration: 1 μg/mL) at days 0 and 3. For inhibition of TGF-β receptor type I signaling under inflammatory conditions, specimens were treated with LPS and 10 μM SB-505124 (Sigma-Aldrich) at day 0 and 3. At day 7, conditioned medium was collected and stored at −80 °C until further analysis.

### 4.4. MTT Staining

After explant culture, specimens were gently rinsed in PBS and incubated in staining solution (50 μg/mL MTT in sterile PBS) at 37 °C for one hour. Samples were photographed at a digital 3D microscope (DVM6, Leica, Wetzlar, Germany) at a magnification of 52×.

### 4.5. Enzyme-Linked Immunosorbent Assay (ELISA)

Secreted protein levels of human pro-collagen-I α1, interleukin-6 and monocyte chemoattractant protein 1 were determined by commercial ELISA kits (Abcam, Bristol, UK, ab210966, ab178013 and ab178886) according to the manufacturer’s instructions. Protein levels were normalized to the wet weight of explanted samples and expressed as pg/mg tissue.

### 4.6. Statistical Analysis

Statistical analyses were performed using GraphPad Prism (v6.2, Graphpad Software Inc., San Diego, CA, USA). Data followed a normal distribution and are reported as means ± SEM. Significant differences were calculated using ratio paired *t*-test or one-way ANOVA. *p*-values less than 0.05 were considered significant.

## Figures and Tables

**Table 1:** Weight-normalized basal secreted protein levels of osteal controls and osteoarthritic knee and facet joint osteochondral specimens.

Secreted Protein	Total OA (*n =* 12)	Facet OA (*n =* 6)	Knee OA (*n =* 6)	Osteal Tissue (*n =* 5)
pro-Col-I (pg/mg)	1273 ± 287	1660 ± 522	886 ± 172	7392 ± 3604 ^†^
IL-6 (pg/mg)	163 ± 45	223 ± 83	102 ± 22	1970 ± 1368 ^†^
MCP-1 (pg/mg)	33 ± 13	49 ± 23	17 ± 4	437 ± 287 ^†^

^†^*p* < 0.05 compared with OA osteochondral specimens by ANOVA; OA, Osteoarthritis.

**Table 2:** Weight-normalized secreted protein levels under inflammatory conditions in the presence and absence of TGF-β receptor type I signaling inhibitor.

Secreted Protein	Treatment	Total OA (*n =* 12)	Facet OA (*n =* 6)	Knee OA (*n =* 6)	Osteal Tissue (*n =* 5)
pro-Col-I (pg/mg)	LPS	1111 ± 432	1604 ± 834	618 ± 165	5460 ± 2306
	LPS + TGF-βRI	327 ± 39 ^‡^	278 ± 51 ^†^	377 ± 55 ^†^	2536 ± 1183
IL-6 (pg/mg)	LPS	652 ± 247	925 ± 471	379 ± 132	2952 ± 1620
	LPS + TGF-βRI	274 ± 82 ^‡^	351 ± 157 ^‡^	196 ± 48 ^†^	5716 ± 4733
MCP-1 (pg/mg)	LPS	80 ± 30	125 ± 55	34 ± 3	1032 ± 680
	LPS + TGF-βRI	139 ± 40 ^†^	200 ± 74	79 ± 7 ^‡^	1223 ± 819

^†^*p <* 0.05, ^‡^ *p <* 0.01 versus LPS group by ratio paired *t*-test.

## Abbreviations

DMOAD	Disease-modifying osteoarthritic drug
OA	Osteoarthritis
LPS	Lipopolysaccharide
TGF-β	Transforming growth factor-β
